# Accuracy of Digital and Conventional Implant Impressions in Edentulous Jaws: A Systematic Review and Meta-Analysis of In Vitro Studies

**DOI:** 10.3390/dj14050304

**Published:** 2026-05-15

**Authors:** Boldizsár László Vánkos, Xinyi Qian, Kata Kelemen, Boglárka Lilla Szentes, Gergely Agócs, Gábor Varga, Péter Hegyi, Péter Hermann, Barbara Kispélyi

**Affiliations:** 1 Department of Prosthodontics, Semmelweis University, 1085 Budapest, Hungary; vankos.boldizsar@semmelweis.hu (B.L.V.); kelemen.kata@semmelweis.hu (K.K.); hermann.peter@semmelweis.hu (P.H.); 2 Centre for Translational Medicine, Semmelweis University, 1085 Budapest, Hungary; szentesboglarka@gmail.com (B.L.S.); agocs.gergely@semmelweis.hu (G.A.); varga.gabor@semmelweis.hu (G.V.); hegyi.peter@semmelweis.hu (P.H.); 3 Department of Biophysics and Radiation Biology, Semmelweis University, 1085 Budapest, Hungary; 4 Department of Oral Biology, Semmelweis University, 1085 Budapest, Hungary; 5 Institute of Pancreatic Diseases, Semmelweis University, 1085 Budapest, Hungary

**Keywords:** dental impression technique, implant prosthodontics, intraoral scanning, stereophotogrammetry, trueness, precision

## Abstract

**Objectives**: Digital impression-taking techniques are widely used due to their many advantages. However, the accuracy of intraoral scanning in full-arch cases remains a matter of debate. The reliability of different digital implant impression techniques remains questionable in completely edentulous, full-arch cases. This review investigated the accuracy of digital implant impression techniques for full-arch use. **Methods**: Our study protocol was registered in the PROSPERO database (CRD42023393091). Data reporting was based on the PRISMA (Preferred Reporting Items for Systematic Reviews and Meta-Analyses) 2020 guidelines, by the Cochrane Handbook. Comparative in vitro studies matching the PICO framework were included. A systematic search was conducted in four databases: PubMed (MEDLINE), EMBASE, Web of Science and the Cochrane Library (Cochrane Central Register of Controlled Trials [CENTRAL]). **Results**: Thirty-four papers were included in analyzing trueness and precision of root mean square (RMS) deviations. No significant differences were observed between test groups. The overall RMS trueness deviation was 53.36 μm [18.44; 88.28] in the extraoral stereophotogrammetry group, 73.88 μm [36.68; 111.09] in the conventional impression group, 99.54 μm [56.22; 142.86] in the IOS plain group, 104.88 μm [36.86; 172.90] in the IOS scanaid group, and 65.58 μm [5.24; 125.92] in the IOS splinted group. Substantial heterogeneity was observed across studies (I^2^ ≈ 100%) **Conclusions**: In case of completely edentulous jaws restored with four to eight implants, digital techniques showed comparable accuracy to the conventional method with no statistically significant differences detected.

## 1. Introduction

Precise transfer of implant positions is critical for the long-term success of implant-supported restorations, particularly in full-arch rehabilitations. Inaccuracies during impression-taking can lead to prosthetic misfit, which has been associated with biological complications (e.g., peri-implantitis) and mechanical failures (e.g., screw loosening, component fractures) [1]. Despite their widespread use, conventional impressions are technique-sensitive. They can be affected by factors such as the dimensional stability of materials, implant angulation, and patient discomfort, particularly in full-arch cases [2].

Intraoral scanners (IOS) are now frequently used for capturing implant positions, offering a contact-free and streamlined approach. However, their accuracy in full-arch, edentulous implant cases remains debated due to the lack of anatomical landmarks, potential stitching errors, and increased scanning distances [3]. Recent systematic reviews and experimental studies have reported inconsistent findings regarding the accuracy of intraoral scanning in full-arch implant cases, particularly in edentulous arches where the lack of anatomical landmarks increases stitching errors and cumulative deviations [4,5,6].

Extraoral stereophotogrammetry has emerged as a promising digital technique to address the limitations of both conventional impression-taking and intraoral scanning. This method involves capturing the 3D spatial relationship of implants using special scanbodies and extraoral cameras, offering significant time-saving and reproducibility advantages in clinical settings [7,8].

Despite the growing body of recent literature, including several comparative in vitro and systematic reviews, a clear consensus has not yet been established regarding the optimal impression technique for full-arch implant-supported restorations.

Comparative studies have yielded mixed results, with some favoring conventional methods for their reliability and others highlighting the potential of digital techniques, especially stereophotogrammetry, in achieving high accuracy [4,9,10].

This systematic review and meta-analysis aimed to evaluate and compare the accuracy of conventional impression-taking, intraoral scanning, and extraoral stereophotogrammetry for implant-supported restorations in edentulous models.

## 2. Materials and Methods

### 2.1. Reporting

Our study protocol was registered in advance using the PROSPERO database [11] (ID CRD42023393091). Reporting in this review adheres to the Preferred Reporting Items for Systematic Reviews and Meta-Analyses (PRISMA) 2020 guidelines as it is documented in Supplementary File S1, PRISMA Checklist and follows the methodological recommendations of the Cochrane Handbook [12].

### 2.2. Eligibility Criteria

Comparative in vitro studies that aligned with the predefined PICO framework were included. The population consisted of dental typodonts with implants; interventions/comparators were, respectively, intraoral scanning with plain scanbodies, intraoral scanning with splinted scanbodies, intraoral scanning with additional scanaids, extraoral stereophotogrammetry, and conventional impression-taking. The primary outcomes assessed were root mean square deviations. Case reports, animal studies, meta-analyses, reviews, and studies utilizing quadrant scans or geometric models were excluded.

### 2.3. Search Strategy

A systematic literature search was carried out on 15 May 2025, using four databases: PubMed (MEDLINE), EMBASE, Web of Science and the Cochrane Library (Cochrane Central Register of Controlled Trials [CENTRAL]). Only articles published in English were considered, and the reference lists of relevant articles and reviews were also screened. No search filters were applied. The search keys used in each database are available in the Supplementary File S4, Search key created for each databases.

After screening and selection, citation chasing was performed to identify potentially relevant studies from the reference lists of the selected full-text articles.

### 2.4. Study Selection and Data Extraction

Duplicate removal and study selection were carried out by two independent reviewers using EndNote X9.3.3 (Clarivate Analytics; Philadelphia, PA, USA) and the Rayyan web-based selection tool [13]. Initial screening was performed on titles and abstracts, followed by full-text selection. Any disagreements were resolved through discussion with a third reviewer. The PRISMA flowchart documents the selection process as presented in Supplementary File S2, Flowchart of Selection. A data extraction sheet was developed within Excel (Microsoft Corp; Redmond, WA, USA) with the help of methodological, statistical, and clinical experts. From each included study, the following information was collected: first author, year of publication, country of origin, study design, population details, key findings, details of interventions, and reported outcomes.

### 2.5. Synthesis Methods

All statistical analysis was carried out in R (R Foundation for Statistical Computing in Vienna, Austria R, v4.4.1) using the following packages: metafor [14], meta [15], and dmetar [15].

The sample size, mean, and the standard deviation (SD) were extracted directly or indirectly from each study. A random-effects model with a generic inverse variance method was used to pool the data. To estimate the heterogeneity variance measure (tau squared), the restricted maximum-likelihood estimator was used with the Q profile method for confidence intervals [16,17].

From the various levels of subdivision of study arms and outcomes, the final choice was based on clinical and statistical (i.e., availability and structure of data) aspects.

When multiple devices or techniques were evaluated within the same study on the same sample, only one was included in the quantitative synthesis to avoid statistical dependency. The selection was based on a ranking list created by methodological and clinical experts in the field, including three independent prosthodontist specialists and researchers with more than fifteen years of experience in IOS related research, relying on reported accuracy outcomes and clinical relevance (e.g., commonly used technologies). However, this approach was not based on a formal consensus methodology and may introduce selection bias.

To create a preference list of the intraoral scanners and impression-taking methods, several factors were considered, including the clinical commonness of use and accuracy.

### 2.6. Risk of Bias (RoB) Assessment

Risk of bias (RoB) was assessed independently by two authors using the QUIN tool (Quality Assessment Tool for In Vitro Studies) [18], which is specifically designed for evaluating RoB in in vitro dental research. The tool consists of a 12-item checklist with predefined scoring criteria: domains clearly described in the article receive two points, those insufficiently described receive one point, and domains not addressed receive zero points. Items deemed not applicable to the study are excluded from scoring.

## 3. Results

### 3.1. Study Selection

Initially, with the systematic search, 4585 articles were identified. Following the duplicate removal, 2588 records remained for screening. Based on the title and abstract, 174 publications were selected for full-text review. Of these, 140 were excluded according to the predefined eligibility criteria. Ultimately, 34 studies met the inclusion criteria and were included in the systematic review and the meta-analysis. The inter-rater agreement was 0.89 for title and abstract screening, and 1 for full-text assessment.

### 3.2. Study Characteristics

The included articles were comparative in vitro studies using experimental gypsum or resin models as reference. The reference model contained four implants in 16 cases [19,20,21,22,23,24,25,26,27,28,29,30,31,32,33,34], five implants in 4 instances [5,9,35,36], six implants in 12 cases [4,37,38,39,40,41,42,43,44,45,46,47], and eight implants in 2 cases [48,49]. The implants were parallel to each other in 17 cases [4,20,21,24,25,26,30,31,33,34,35,38,43,44,45,47,48], angulated in 16 instances [9,19,20,21,22,23,24,27,29,32,35,36,37,39,46,49], and it was not disclosed in 5 cases [5,28,40,41,42]. In four articles, models with both angulated and parallel implants were examined [20,21,24,35]. Additional characteristics like angulation of the implants, level of impression-taking, method of impression-taking and so on were too heterogeneous to create subgroups, but are included in the plots as “comments”.

For reference scanning, 27 articles used laboratory-grade desktop scanners, three studies used industrial-grade scanners, and four studies used a coordinate-measuring machine.

### 3.3. Outcome Assessment

The accuracy outcomes were assessed by measuring the surface of the models or the implant analogs in the reference and test models. The articles mentioned three methods: a coordinate-measuring machine (CMM), a laboratory-grade desktop scanner, or an industrial-grade scanner.

Based on the results of Borbola et al. and Yilmaz et al., scans performed by laboratory-grade scanners can be alternatives to those of reference industrial scanner. Moreover, some laboratory-grade scanners might be used as reference scanners for studying IOS accuracy [50,51].

The definitive models were scanned with scanbodies attached to the analogs, and the deviations were measured by superimposing the reference and test standard tessellation (STL) files.

### 3.4. Accuracy Outcomes—RMS Trueness

The root mean square (RMS) trueness deviations of the models were reported overall in eighteen studies. The means and standard deviations were pooled together based on the acquisition method according to the following: extraoral stereophotogrammetry, conventional impression, IOS plain, IOS scanaid, IOS splinted.

**Figure 1:**
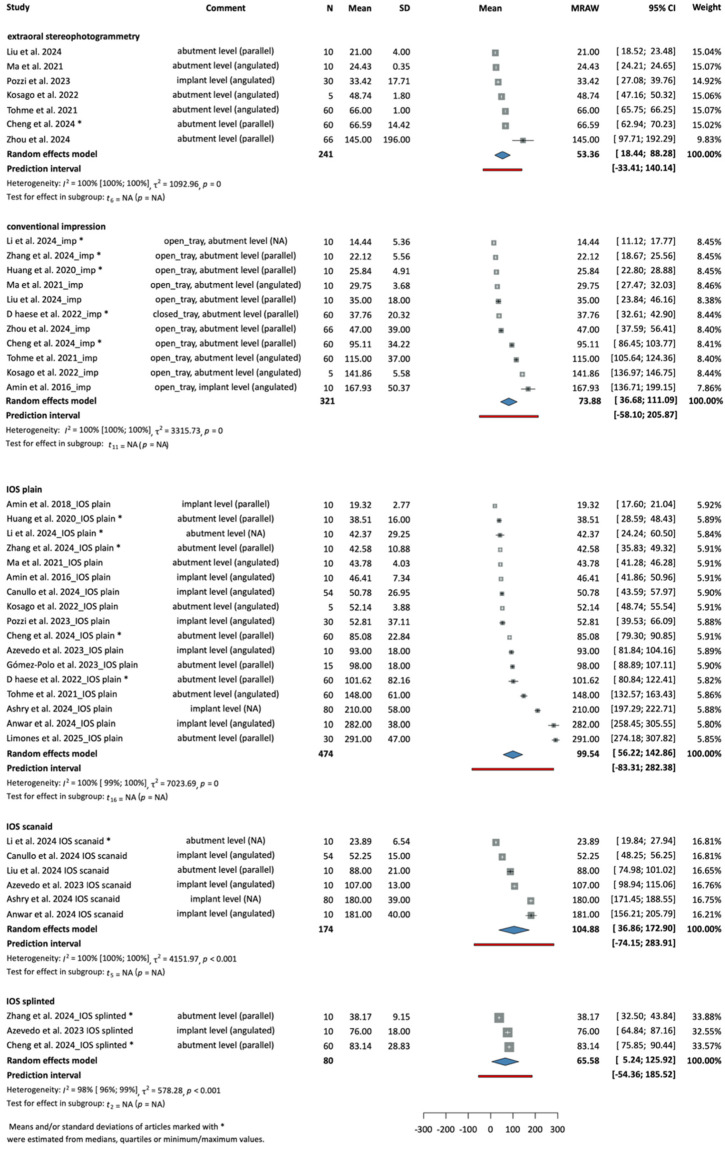
Forest plot of the RMS trueness deviations measured in the tested groups subgrouped by the impression-taking method (µm) [4,19,21,23,26,27,28,29,30,31,35,36,37,38,42,43,44,46].

**Figure 2:**
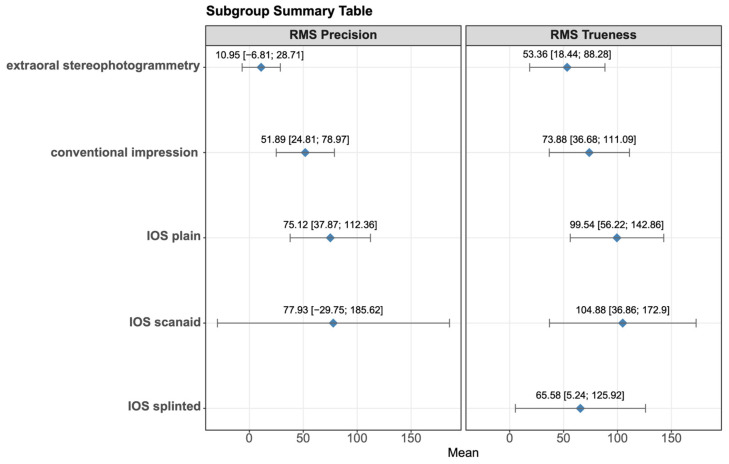
Subgroup summary plot of the impression-taking methods (µm).

Figure 1 shows the RMS trueness values of the interventions grouped by method, respectively. Extraoral stereophotogrammetry showed an overall mean of 53.36 µm with a 95% confidence interval of [18.44; 88.28], conventional impression showed an overall mean of 73.88 µm [36.68; 111.09], IOS plain showed an overall mean of 99.54 µm [56.22; 142.86], IOS scanaid showed an overall mean of 104.88 µm [36.86; 172.9] and IOS splinted showed an overall mean of 65.58 µm [5.24; 125.92]. Figure 2 represents the summed results of each subgroup, providing a more straightforward and visual view.

### 3.5. Accuracy Outcomes—RMS Precision

The root mean square (RMS) precision deviations of the models were reported overall in twelve studies. The means and standard deviations were pooled together based on the acquisition method according to the following: extraoral stereophotogrammetry, conventional impression, IOS plain, IOS scanaid, IOS splinted. In the IOS splinted category, only two articles were included, so no random effect model was calculated; however, the studies are included in the figure to show the tendency.

**Figure 3:**
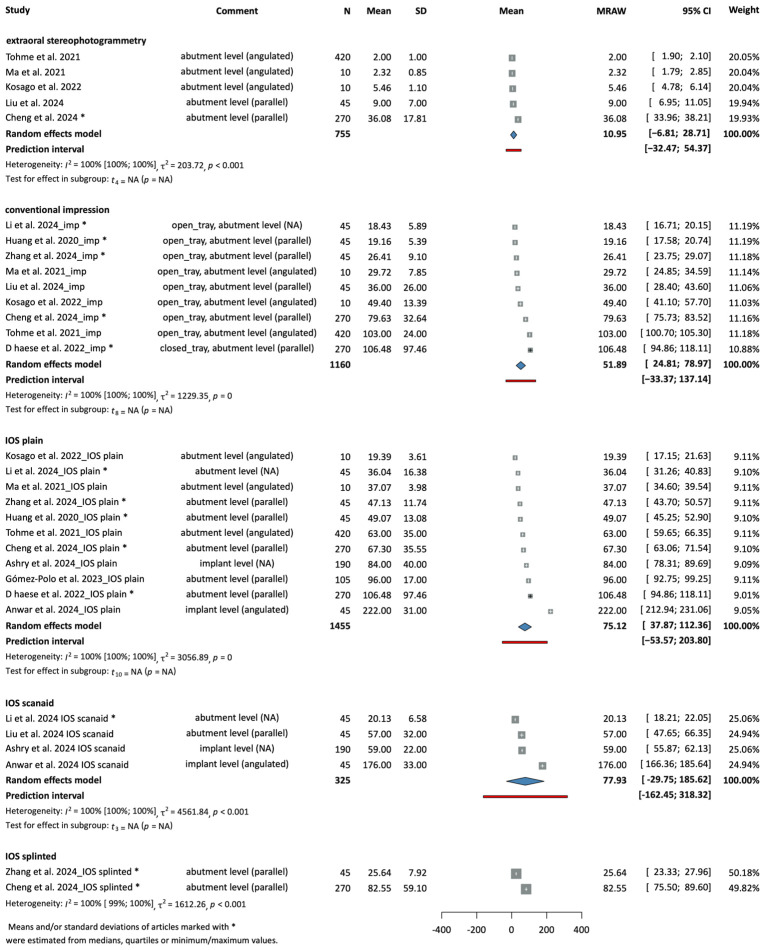
Forest plot of the RMS precision deviations measured in the tested groups subgrouped by the impression-taking method (µm) [4,23,26,27,28,30,36,37,38,42,44,45].

Figure 3 shows the RMS precision values of the interventions grouped by method, respectively. Extraoral stereophotogrammetry showed an overall mean of 10.95 µm [−6.81; 28.71], conventional impression showed an overall mean of 51.89 µm [24.81; 78.97], IOS plain showed an overall mean of 75.12 µm [37.87; 122.36], and IOS scanaid showed an overall mean of 77.93 µm [−29.75; 185.62]. Figure 2 represents the summed results of each subgroup, providing a more straightforward and visual view.

### 3.6. Risk of Bias

All included articles were assessed for risk of bias (RoB) using the 12-item checklist of QUIN RoB tool, and categorized as having low, moderate, or high RoB. The summary of RoB can be seen in Supplementary File S3, Summary of RoB. A total of 22 studies were categorized as “some concerns” and 12 studies were judged to present “low risk” of bias. In most cases, the distinction from low to medium risk was due to missing or inaccurate sample size calculations and a lack of details regarding the number and experience of operators and assessors. No studies were excluded based on a high RoB rating.

## 4. Discussion

It was hypothesized that digital impression-taking technologies (including intraoral scanning and extraoral stereophotogrammetry) would be less accurate than the gold-standard conventional impression-taking method. No statistically significant differences were found between the investigated methods, indicating that our original hypothesis that digital impression-taking technologies are less accurate is not confirmed.

Previously, most intraoral scanners were not recommended for full-arch cases [52], and still to date, it is questionable whether they can produce high enough accuracy for full-arch restorations [53]. This is especially true in cases of complete edentulousness and implant restorations with large interimplant distances. Intraoral scanners stitch images together based on overlapping areas and identical points to create a 3D mesh. The more details a surface has, the more reference points a scanner can find. For example, the occlusal surface of premolars and molars provides sufficient reference, but in large edentulous areas, the lack of stable reference points makes the stitching process difficult. Scanning is especially challenging in cases of completely edentulous lower jaws, where even the mucosa is moving.

To understand and interpret accuracy results in the context of their clinical relevance, a threshold of clinically acceptable accuracy should be taken as a baseline. Determining clinical relevance is difficult since there is no clear consensus regarding the clinically acceptable accuracy of prostheses or dental models [54]. In the past decades, based on the results of Mclean et al. [55], 120 µm was considered the threshold of clinically acceptable accuracy. Although this article estimates cement film thickness, not implant positional accuracy, it is often cited in accuracy-related studies.

In modern dentistry and prosthodontics, these results seem to be already far exceeded. However, it is important to distinguish between impression accuracy, prosthetic fit, and clinically acceptable misfit. While RMS deviations reflect impression accuracy, prosthetic fit depends on multiple downstream factors, and clinically acceptable misfit thresholds are derived from different biological and mechanical considerations. Therefore, caution is needed when extrapolating accuracy values to clinical acceptability.

Schwindling et al. reported a misfit as low as 36–88 µm in monolithic full-arch restorations using digital workflow within in vitro conditions [56]. In this article, full-arch implant restorations were fabricated; thus, the relevance of their results to our study is more significant. However, it must be noted that the fit of the prostheses, and not the position of implants, was measured. According to the results of Lyu et al., monolithic zirconia crowns manufactured with nanoparticle jetting can have RMS deviations as low as 22.9 µm [57].

Accepting the often-cited threshold of 120 µm as a baseline of acceptance means anything under that value should be deemed acceptable. Considering this, all the methods investigated in our article are equally acceptable, since their mean trueness deviations range from 53.36 (18.44; 88.28) to 104.88 (36.86; 172.90) and mean precision deviations range from 10.95 (−6.81; 28.71) to 77.93 (−29.75; 185.62). It is essential to note that the difference between the best (extraoral stereophotogrammetry) and worst (IOS scanaid) methods is almost double.

Katsoulis et al. investigated the effect of misfit on the biological and mechanical complications of screw-retained implant-supported, fixed dentures. Screw-related mechanical complications were observed; however, they did not find sufficient evidence for correlation with the degree of misfit [58].

The results of Abdelrehim et al. suggest that mechanical consequences are more critical than biological. Researchers determined a high range of tolerable misfit; however, they were unable to provide sufficient evidence for a universal threshold for clinical acceptability. Neither horizontal nor vertical misfit values of 150 µm in their study caused mechanical adverse effects. They found that a misfit up to 1 mm is still tolerable when considering biological failures [59].

Several new scanning methods and products have been introduced to aid the difficulties associated with full-arch (especially edentulous) scanning, thus reducing inaccuracies. One of these methods is splinting the scanbodies similar to conventional impression copings. For this purpose, dental floss and self-curing resin or prefabricated splints can be used [4,30,60]. Splinting impression copings for conventional impressions has a mechanical purpose: holding and securing the copings within a rigid, stable framework to prevent the movement of the copings [61,62,63]. For intraoral scanning, it provides reference points between the scanbodies, thus aiding the stitching process in edentulous areas. Splinting the scanbodies resulted in notably better trueness and precision values than “plain” intraoral scanning (scanning without splinting or using any additional scanning aid). However, it must be noted that the subgroup of splinted scanbodies only contained three articles in trueness and two articles in the precision outcomes.

Studies in the subgroup “scanaid” used different ways of compensating inaccuracies: longitudinally extended scanflags [42], prefabricated scanbody attachments [28,44], uniquely modified scanbodies [27], and geometric attachments inserted onto the mucosa [27]. These methods or devices (all pooled together) did not seem to enhance intraoral scanning; and produced less accurate results than “plain” scanning. However, in this case, the difference was truly minimal (5.34 µm in trueness and 2.81 µm in precision). A low study number and extremely high heterogenity in the group must be considered a limitation; therefore, the clinical value cannot be clearly stated.

Extraoral stereophotogrammetry cameras are known to be highly accurate, which has also been confirmed by our results. Trueness and precision values showed the highest accuracy within all groups. These systems, however, are costly—they are limited to one specific purpose, and they require the use of an intraoral scanner to capture the mucosa, nonetheless. These systems also require all the scanbodies to be visible at the same time, which might cause difficulties in unfavorable anatomical situations or with patients who have limited mouth opening.

In our analysis, notable differences occurred between the accuracy of the conventional and different digital implant impression-taking methods. Although the differences are not statistically significant, clinical consequences cannot be ruled out. An exact accuracy threshold could be determined by assessing the implications of an implant restoration misfit in relation to the degree of deviation.

The studies included in our analysis were conducted in vitro, thus compromising the clinical relevance of the results. Clinical parameters such as saliva, temperature, material mixing, and ambient lighting can affect impression-taking accuracy.

A key finding of this meta-analysis is the extremely high heterogeneity observed across the included studies (I^2^ ≈ 100%). This level of heterogeneity reflects substantial variability in study designs, measurement methods, reference devices, implant configurations, and data processing approaches. Consequently, the pooled estimates should be interpreted with caution, as they may not represent a single underlying effect. The appropriateness of quantitative pooling in this context is therefore limited, and the results should primarily be considered as exploratory rather than definitive.

The included articles measured the deviations with digital methods, thus reporting exact numerical data, allowing us to conduct statistical analysis. In vivo studies often rely on clinical data, such as passivity of fit or X-ray imaging. Our analyses show high heterogeneity, possibly because of the included studies’ differences in methodology, data analysis, and reporting.

### 4.1. Strengths

Our study provides several methodological and clinical contributions beyond previous reviews. First, it includes a large number of recent in vitro studies reflecting current implant impression technologies and digital workflows.

Second, multiple impression techniques—including intraoral scanning, conventional impressions, and extraoral stereophotogrammetry—were analyzed simultaneously within a single meta-analytic framework. This allows for direct comparison across methods that are often evaluated separately in the literature.

Third, the use of standardized root mean square (RMS) values for both trueness and precision enabled consistent quantitative synthesis across heterogeneous studies.

Finally, intraoral scanning techniques were further subdivided into clinically relevant categories (plain, splinted, and scanaid), providing a more detailed and practice-oriented evaluation than most previous analyses.

### 4.2. Limitations

The main limitation of our review was the heterogeneity of the included studies regarding the specific methods of conventional and digital impression-taking. Including only in vitro studies limits the information value for clinicians. In many cases, the RoB showed concerns, most often related to the lack of sample size calculation.

Another limitation is the selection of a single method from studies reporting multiple devices. Although this was done to avoid statistical dependency, the selection process was partly based on expert judgment and may introduce selection bias.

Restriction to English-language publications may have introduced language bias.

## 5. Conclusions

Intraoral scanning technologies showed comparable accuracy to the conventional impression-taking methods within in vitro settings. No statistically significant differences were observed in the case of completely edentulous jaws restored with four to eight implants.

Although no statistically significant differences were found, stereophotogrammetry showed the most favorable numerical values.

## 6. Clinical Implications

In clinical practice, intraoral scanning may be preferred for patient comfort and workflow efficiency, while conventional impressions remain a reliable and well-known option in challenging full-arch cases. Stereophotogrammetry may be advantageous when maximum accuracy is required and resources are available.

## 7. Implications for Research

Future studies should prioritize well-designed clinical trials to validate these in vitro findings. Standardized methodologies and reporting are urgently needed to reduce heterogeneity, and clinically meaningful accuracy thresholds must be established by linking deviation values to biological and mechanical outcomes. Additionally, comparative evaluation of digital workflows, including stereophotogrammetry, under real in vivo clinical conditions is essential.

## Data Availability

The raw data supporting the conclusions of this article will be made available by the authors on request.

## Supplementary Materials

The following supporting information can be downloaded at https://www.mdpi.com/article/10.3390/dj14050304/s1, Supplementary File S1. PRISMA Checklist. Supplementary File S2. PRISMA Flowchart of selection. Supplementary File S3. Summary of RoB. Supplementary File S4. Search key created for each databases.
