# Supplementary material for: Accuracy of Digital and Conventional Implant Impressions in Edentulous Jaws: A Systematic Review and Meta-Analysis of In Vitro Studies
Source: Dent J (Basel). 2026 May 15;14(5):304. doi: 10.3390/dj14050304 (PMC13206515; doi:10.3390/dj14050304)
Supplement: Supplementary file 1 [file dentistry-14-00304-s001.zip › File S3 Summary of RoB.pdf]

Criteria No.	Criteria	Details		Adequately specified (Score=2)	Inadequately specified (Score=1)	Not specified (Score=0)	Not applicable		Score (Total score×100)/ (2×number of criteria applicable)	>70% low risk, 50-70% meidum risk, <50%high risk
1	Clearly stated aims/objectives	Study should clearly state aims and/ or objectives, which should then be followed throughout.								
2	Detailed explanation of sample size calculation	Details regarding method by which given sample size calculated should be clearly stated.<br>Details regarding software program, formula, and parameters used for calculation of sample size should also be specified.								
3	Detailed explanation of sampling technique	Details regarding predefined population from which sample has been selected.<br>Details of sampling technique and inclusion and exclusion criteria should be clearly stated.								
4	Details of comparison group	Details of comparison group (positive control, negative control, or standard) should be clearly specified.								
5	Detailed explanation of methodology	Clarity of procedure, method of standardization, and details of any universal standards used (if applicable) should be clearly stated.								
6	Operator details	Number of operators and details regarding training and calibration of operator/s (inter-operator and intra- operator reliability) should be clearly specified.								
7	Randomization	Details regarding sequence generation and allocation concealment should be clearly stated.								
8	Method of measurement of outcome	Clarity of procedure and rationale for choosing method should be stated.<br>Method of standardization along with details of any universal standards used (if applicable) should also be clearly specified.								
9	Outcome assessor details	Number of outcome assessors and details regarding training and calibration of assessor/s (inter- outcome and intra-outcome assessor reliability) should be clearly specified.								
10	Blinding	Details regarding blinding of operator(s), outcome assessor(s), and statistician should be clearly specified.								
11	Statistical analysis	Details regarding software program used and statistical analysis should be clearly specified.								
12	Presentation of results	Outcome should be based on predefined aims and/or objectives.<br>All data should be adequately tabulated with baseline data clearly specified (if applicable).								

	Domain 1	Domain 2	Domain 3	Domain 4	Domain 5	Domain 6	Domain 7	Domain 8	Domain 9	Domain 10	Domain 11	Domain 12	Summary
Zhou 2024	Inadequately specified	Not specified	Not applicable	Not applicable	Adequately specified	Inadequately specified	Not applicable	Adequately specified	Not specified	Not applicable	Adequately specified	Adequately specified	medium risk
Zhang 2024	Adequately specified	Not specified	Not applicable	Not applicable	Adequately specified	Inadequately specified	Not applicable	Adequately specified	Inadequately specified	Not applicable	Adequately specified	Adequately specified	low risk
Wu 2024	Adequately specified	Not specified	Not applicable	Not applicable	Adequately specified	Inadequately specified	Not applicable	Adequately specified	Not specified	Not applicable	Inadequately specified	Adequately specified	medium risk
Vandeweghe 2017	Adequately specified	Not specified	Not applicable	Not applicable	Adequately specified	Not specified	Not applicable	Adequately specified	Not specified	Not applicable	Adequately specified	Adequately specified	medium risk
Rajendran 2021	Adequately specified	Not specified	Not applicable	Not applicable	Adequately specified	Inadequately specified	Not applicable	Adequately specified	Inadequately specified	Not applicable	Inadequately specified	Adequately specified	medium risk
Pera 2016	Adequately specified	Not specified	Not applicable	Not applicable	Adequately specified	Not specified	Not applicable	Adequately specified	Not specified	Not applicable	Adequately specified	Adequately specified	medium risk
Liu 2024	Adequately specified	Adequately specified	Not applicable	Not applicable	Adequately specified	Inadequately specified	Not applicable	Adequately specified	Not specified	Not applicable	Adequately specified	Adequately specified	low risk
Limones 2025	Adequately specified	Adequately specified	Not applicable	Not applicable	Adequately specified	Inadequately specified	Not applicable	Adequately specified	Not specified	Not applicable	Adequately specified	Adequately specified	low risk
Li 2024	Adequately specified	Not specified	Not applicable	Not applicable	Adequately specified	Inadequately specified	Not applicable	Adequately specified	Not specified	Not applicable	Adequately specified	Adequately specified	medium risk
Pozzi 2023	Adequately specified	Adequately specified	Not applicable	Not applicable	Adequately specified	Not specified	Not applicable	Adequately specified	Not specified	Not applicable	Adequately specified	Adequately specified	low risk
Papaspyridakos 2015	Adequately specified	Not specified	Not applicable	Not applicable	Adequately specified	Inadequately specified	Not applicable	Adequately specified	Not specified	Not applicable	Adequately specified	Adequately specified	medium risk
Shaikh 2022	Adequately specified	Not specified	Not applicable	Not applicable	Adequately specified	Not specified	Not applicable	Adequately specified	Not specified	Not applicable	Adequately specified	Adequately specified	medium risk
Ma 2021	Inadequately specified	Not specified	Not applicable	Not applicable	Adequately specified	Adequately specified	Not applicable	Adequately specified	Not specified	Not applicable	Adequately specified	Adequately specified	medium risk
Gómez-Polo 2023	Adequately specified	Inadequately specified	Not applicable	Not applicable	Adequately specified	Inadequately specified	Not applicable	Adequately specified	Not specified	Not applicable	Adequately specified	Adequately specified	low risk
Kosago 2022	Adequately specified	Adequately specified	Not applicable	Not applicable	Inadequately specifie	Inadequately specified	Not applicable	Inadequately specifie	Not specified	Not applicable	Inadequately specified	Adequately specified	medium risk
Huang 2020	Adequately specified	Not specified	Not applicable	Not applicable	Adequately specified	Inadequately specified	Not applicable	Adequately specified	Not specified	Not applicable	Inadequately specified	Inadequately specified	medium risk
Drancourt 2023	Adequately specified	Inadequately specified	Not applicable	Not applicable	Adequately specified	Inadequately specified	Not applicable	Inadequately specifie	Not specified	Not applicable	Inadequately specified	Adequately specified	medium risk
Ribeiro 2018	Adequately specified	Adequately specified	Not applicable	Not applicable	Adequately specified	Inadequately specified	Not applicable	Adequately specified	Inadequately specified	Not applicable	Adequately specified	Adequately specified	low risk
Revilla-León 2021	Adequately specified	Not specified	Not applicable	Not applicable	Adequately specified	Not specified	Not applicable	Adequately specified	Not specified	Not applicable	Inadequately specified	Adequately specified	medium risk
Tan 2018	Adequately specified	Not specified	Not applicable	Not applicable	Adequately specified	Inadequately specified	Not applicable	Adequately specified	Not specified	Not applicable	Adequately specified	Adequately specified	medium risk
Tohme 2021	Adequately specified	Inadequately specified	Not applicable	Not applicable	Adequately specified	Inadequately specified	Not applicable	Inadequately specifie	Not specified	Not applicable	Adequately specified	Adequately specified	medium risk
Cheng 2024	Adequately specified	Not specified	Not applicable	Not applicable	Adequately specified	Inadequately specified	Not applicable	Inadequately specifie	Not specified	Not applicable	Adequately specified	Adequately specified	medium risk
Canullo 2024	Adequately specified	Adequately specified	Not applicable	Not applicable	Adequately specified	Not specified	Not applicable	Adequately specified	Not specified	Not applicable	Adequately specified	Adequately specified	low risk
Kim 2017	Adequately specified	Not specified	Not applicable	Not applicable	Adequately specified	Not specified	Not applicable	Adequately specified	Not specified	Not applicable	Adequately specified	Adequately specified	medium risk
Ke 2023	Adequately specified	Not specified	Not applicable	Not applicable	Adequately specified	Inadequately specified	Not applicable	Adequately specified	Not specified	Not applicable	Adequately specified	Adequately specified	medium risk
Gómez-Polo 2022	Adequately specified	Not specified	Not applicable	Not applicable	Adequately specified	Inadequately specified	Not applicable	Inadequately specifie	Not specified	Not applicable	Adequately specified	Adequately specified	medium risk
D'Haese 2022	Adequately specified	Inadequately specified	Not applicable	Not applicable	Adequately specified	Not specified	Not applicable	Adequately specified	Not specified	Not applicable	Adequately specified	Adequately specified	medium risk
Azevedo 2023	Adequately specified	Adequately specified	Not applicable	Not applicable	Adequately specified	Adequately specified	Not applicable	Adequately specified	Inadequately specified	Not applicable	Adequately specified	Adequately specified	low risk

Asli 2021	Adequately specified	Not specified	Not applicable	Not applicable	Adequately specified	Inadequately specified	Not applicable	Adequately specified	Inadequately specified	Not applicable	Inadequately specified	Adequately specified	medium risk
Ashry 2024	Adequately specified	Adequately specified	Not applicable	Not applicable	Adequately specified	Inadequately specified	Not applicable	Adequately specified	Not specified	Not applicable	Adequately specified	Adequately specified	low risk
Anwar 2024	Adequately specified	Not specified	Not applicable	Not applicable	Adequately specified	Inadequately specified	Not applicable	Adequately specified	Not specified	Not applicable	Adequately specified	Adequately specified	medium risk
Amin 2016	Adequately specified	Inadequately specified	Not applicable	Not applicable	Adequately specified	Adequately specified	Not applicable	Inadequately specifie	Not specified	Not applicable	Adequately specified	Adequately specified	low risk
Alikhasi 2018	Adequately specified	Not specified	Not applicable	Not applicable	Adequately specified	Adequately specified	Not applicable	Adequately specified	Adequately specified	Not applicable	Adequately specified	Adequately specified	low risk
Albayrak 2020	Adequately specified	Not specified	Not applicable	Not applicable	Adequately specified	Adequately specified	Not applicable	Adequately specified	Not specified	Not applicable	Adequately specified	Adequately specified	low risk

	scores												Summary		Risk
	Domain 1	Domain 2	Domain 3	Domain 4	Domain 5	Domain 6	Domain 7	Domain 8	Domain 9	Domain 10	Domain 11	Domain 12			
Zhou 2024	1	0	NA	NA	2	1	NA	2	0	NA	2	2	10	62,5	medium risk
Zhang 2024	2	0	NA	NA	2	1	NA	2	1	NA	2	2	12	75	low risk
Wu 2024	2	0	NA	NA	2	1	NA	2	0	NA	1	2	10	62,5	medium risk
Vandeweghe 2017	2	0	NA	NA	2	0	NA	2	0	NA	2	2	10	62,5	medium risk
Rajendran 2021	2	0	NA	NA	2	1	NA	2	1	NA	1	2	11	68,75	medium risk
Pera 2016	2	0	NA	NA	2	0	NA	2	0	NA	2	2	10	62,5	medium risk
Liu 2024	2	2	NA	NA	2	1	NA	2	0	NA	2	2	13	81,25	low risk
Limones 2025	2	2	NA	NA	2	1	NA	2	0	NA	2	2	13	81,25	low risk
Li 2024	2	0	NA	NA	2	1	NA	2	0	NA	2	2	11	68,75	medium risk
Pozzi 2023	2	2	NA	NA	2	0	NA	2	0	NA	2	2	12	75	low risk
Papaspyridakos 2015	2	0	NA	NA	2	1	NA	2	0	NA	2	2	11	68,75	medium risk
Shaikh 2022	2	0	NA	NA	2	0	NA	2	0	NA	2	2	10	62,5	medium risk
Ma 2021	1	0	NA	NA	2	2	NA	2	0	NA	2	2	11	68,75	medium risk
Gómez-Polo 2023	2	1	NA	NA	2	1	NA	2	0	NA	2	2	12	75	low risk
Kosago 2022	2	2	NA	NA	1	1	NA	1	0	NA	1	2	10	62,5	medium risk
Huang 2020	2	0	NA	NA	2	1	NA	2	0	NA	1	1	9	56,25	medium risk
Drancourt 2023	2	1	NA	NA	2	1	NA	1	0	NA	1	2	10	62,5	medium risk
Ribeiro 2018	2	2	NA	NA	2	1	NA	2	1	NA	2	2	14	87,5	low risk
Revilla-León 2021	2	0	NA	NA	2	0	NA	2	0	NA	1	2	9	56,25	medium risk
Tan 2018	2	0	NA	NA	2	1	NA	2	0	NA	2	2	11	68,75	medium risk
Tohme 2021	2	1	NA	NA	2	1	NA	1	0	NA	2	2	11	68,75	medium risk
Cheng 2024	2	0	NA	NA	2	1	NA	1	0	NA	2	2	10	62,5	medium risk
Canullo 2024	2	2	NA	NA	2	0	NA	2	0	NA	2	2	12	75	low risk
Kim 2017	2	0	NA	NA	2	0	NA	2	0	NA	2	2	10	62,5	medium risk
Ke 2023	2	0	NA	NA	2	1	NA	2	0	NA	2	2	11	68,75	medium risk
Gómez-Polo 2022	2	0	NA	NA	2	1	NA	1	0	NA	2	2	10	62,5	medium risk
D'Haese 2022	2	1	NA	NA	2	0	NA	2	0	NA	2	2	11	68,75	medium risk
Azevedo 2023	2	2	NA	NA	2	2	NA	2	1	NA	2	2	15	93,75	low risk
Asli 2021	2	0	NA	NA	2	1	NA	2	1	NA	1	2	11	68,75	medium risk
Ashry 2024	2	2	NA	NA	2	1	NA	2	0	NA	2	2	13	81,25	low risk
Anwar 2024	2	0	NA	NA	2	1	NA	2	0	NA	2	2	11	68,75	medium risk
Amin 2016	2	1	NA	NA	2	2	NA	1	0	NA	2	2	12	75	low risk
Alikhasi 2018	2	0	NA	NA	2	2	NA	2	2	NA	2	2	14	87,5	low risk
Albayrak 2020	2	0	NA	NA	2	2	NA	2	0	NA	2	2	12	75	low risk
