# Supplementary material for: Accuracy of Digital and Conventional Implant Impressions in Edentulous Jaws: A Systematic Review and Meta-Analysis of In Vitro Studies
Source: Dent J (Basel). 2026 May 15;14(5):304. doi: 10.3390/dj14050304 (PMC13206515; doi:10.3390/dj14050304)
Supplement: Supplementary file 1 [file dentistry-14-00304-s001.zip › File S4 Search key created for each databases.pdf]

**The search was conducted using the following search key for PubMed:**

**PubMed**

(implant* AND (full arch OR edentulous OR “all-on-4” OR complete*)) AND

(scan* OR coded OR combined OR photogram* OR impression OR conventional OR “open tray”) AND (accuracy OR trueness OR precision)

**EMBASE**

(implant* AND (full arch OR edentulous OR all-on-4 OR complete*)) AND

(scan* OR coded OR combined OR photogram* OR impression OR conventional OR open tray) AND (accuracy OR trueness OR precision)

**CENTRAL**

(implant* AND (full arch OR edentulous OR all-on-4 OR complete*)) AND

(scan* OR coded OR combined OR photogram* OR impression OR conventional OR open tray) AND (accuracy OR trueness OR precision)

**Web of Science**

(implant* AND (implant OR prosthodontic*)) AND

(scan* OR “coded” OR “combined” OR photogram* OR “impression” OR “conventional” OR “open tray”) AND

(“full arch” OR “edentulous” OR “all-on-4” OR complete*) AND (“accuracy” OR “trueness” OR “precision”)
