# Supplementary figures and images for: Accuracy of Digital and Conventional Implant Impressions in Edentulous Jaws: A Systematic Review and Meta-Analysis of In Vitro Studies
Source: Dent J (Basel). 2026 May 15;14(5):304. doi: 10.3390/dj14050304 (PMC13206515; doi:10.3390/dj14050304)

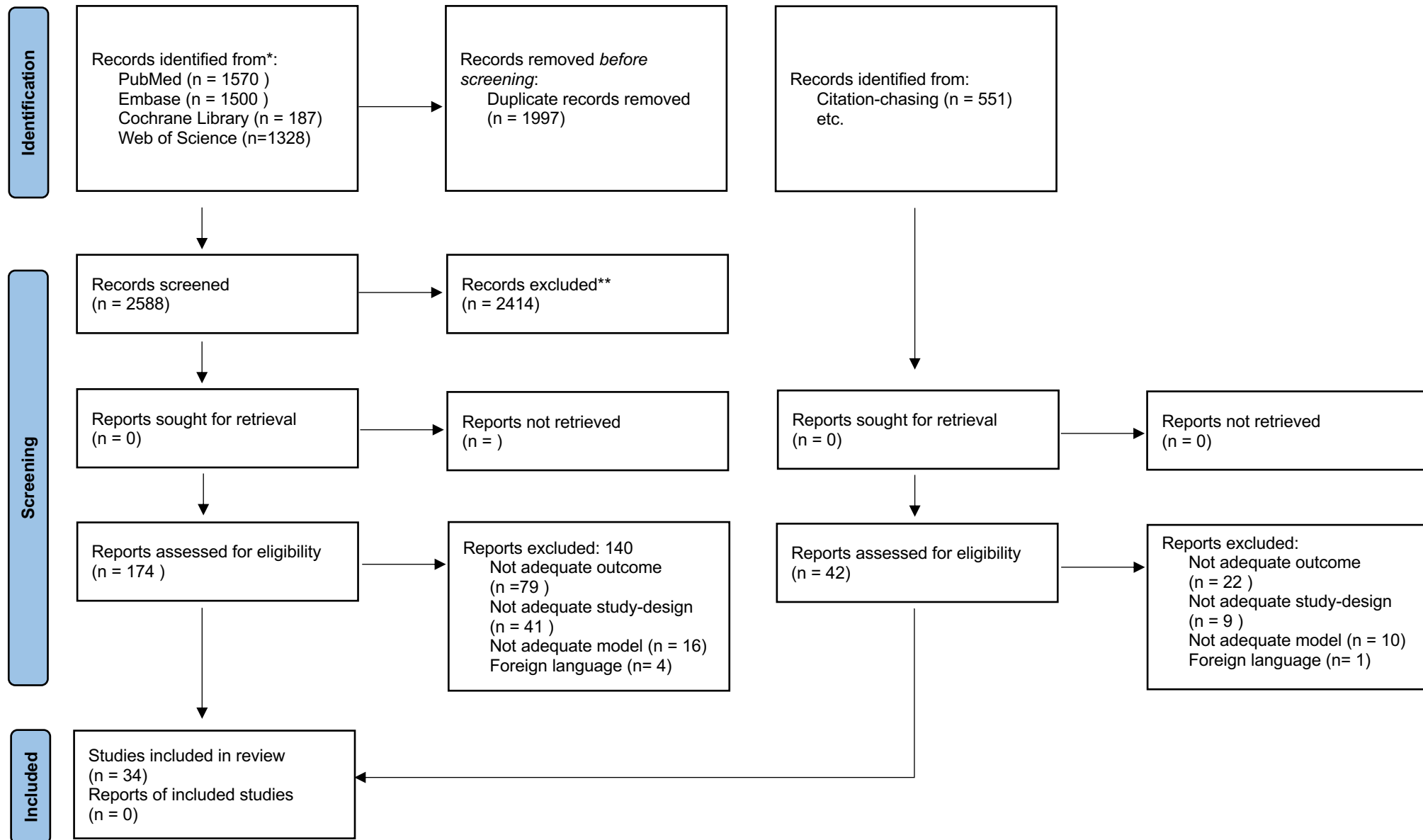

Supplement: Supplementary file 1 [file dentistry-14-00304-s001.zip › File S2 PRISMA flowchart of selection.pdf]
